# *HER2*-encoded mir-4728 forms a receptor-independent circuit with miR-21-5p through the non-canonical poly(A) polymerase PAPD5

**DOI:** 10.1038/srep35664

**Published:** 2016-10-18

**Authors:** Inga Newie, Rolf Søkilde, Helena Persson, Thiago Jacomasso, Andrej Gorbatenko, Åke Borg, Michiel de Hoon, Stine F. Pedersen, Carlos Rovira

**Affiliations:** 1 Department of Clinical Sciences, Lund, Division of Oncology and Pathology, Lund University Cancer Center, Lund, Sweden; 2 BioCARE, Strategic Cancer Research Program, Lund, Sweden; 3 Department of Biology, University of Copenhagen, Copenhagen, Denmark; 4 CREATE Health, Strategic Centre for Translational Cancer Research, Lund, Sweden; 5 Division of Genomic Technologies, RIKEN Center for Life Science Technologies, Yokohama, Japan

## Abstract

We previously reported that the human *HER2* gene encodes the intronic microRNA mir-4728, which is overexpressed together with its oncogenic host gene and may act independently of the HER2 receptor. More recently, we also reported that the oncogenic miR-21-5p is regulated by 3′ tailing and trimming by the non-canonical poly(A) polymerase PAPD5 and the ribonuclease PARN. Here we demonstrate a dual function for the *HER2* locus in upregulation of miR-21-5p; while HER2 signalling activates transcription of mir-21, miR-4728-3p specifically stabilises miR-21-5p through inhibition of PAPD5. Our results establish a new and unexpected oncogenic role for the *HER2* locus that is not currently being targeted by any anti-HER2 therapy.

The human epidermal growth factor receptor 2, *HER2* or *ERBB2* (hereafter called *HER2*) is a well-characterised oncogene and amplification and/or protein overexpression of HER2 is common in human cancers of the breast, ovary, lung, stomach, colon, pancreas, and endometrium (for a review see ref. 1). In breast cancer, *HER2* amplification is observed in 15–20% of cases and is associated with more aggressive tumour behaviour^2^. Breast cancer patients with tumours overexpressing HER2 (HER2-positive tumours) are selected for targeted therapy against the receptor. The development of anti-HER2 therapy that started with the monoclonal antibody trastuzumab (Herceptin), which binds the extracellular domain of HER2, has revolutionised the management of HER2-positive breast cancer and become a paradigm for personalised medicine. This treatment option is today also indicated for treatment of HER2-positive metastatic gastric cancer.

However, global gene expression analysis has shown that HER2-positive tumours constitute a heterogeneous group^3^ and, despite the success, many HER2-positive tumours escape the effects of HER2 inhibition and a large portion of initial responders relapse. Alternative treatment options have been developed to overcome this important clinical problem. These include antibodies that target other regions of the extracellular domain (pertuzumab), protein tyrosine kinase inhibitors (lapatinib, neratinib, afatinib), and inhibitors that block HER2 signalling. All therapeutic routes are directed against the function of the receptor since it is assumed to be the only oncogenic unit encoded in the locus. Activation of membrane-bound HER2 recruits signal transducers that control cell proliferation, differentiation, migration and apoptosis but HER2 can also modulate expression of post-transcriptional regulators such as miRNAs. HER2 signalling controls expression of the oncogenic miR-21-5p and HER2 overexpression has been shown to induce mir-21 transcription in normal and cancer cells^4^, either directly or via transcriptional activation of the neighbouring *VMP1* gene that can bypass polyadenylation signals to include the mir-21 precursor^5^.

We identified a miRNA gene, *mir-4728*, that is encoded in an intron of the *HER2* gene, indicating that, contrary to what is commonly assumed, the *HER2* locus has a second role in addition to the membrane receptor^6^. *HER2* transcription simultaneously produces *HER2* mRNA as well as mir-4728 to the point that expression of mir-4728 has been suggested to accurately mark HER2 status and to work as a non-invasive biomarker in HER2-positive breast and gastric cancer^7^. Among other functions, miR-4728-3p has been shown to modulate expression of oestrogen receptor alpha (ERα)^8^ and may act as a negative feedback mechanism for HER2 signalling by regulating the MAPK pathway^9^.

In collaboration with the de Hoon laboratory we have also reported that the non-canonical poly(A) polymerase PAPD5 is involved in non-templated 3′ adenylation of miRNAs^10^. In particular, PAPD5 adenylates the 3′ end of miR-21-5p, marking it for 3′ -to-5′ trimming by the poly(A) specific ribonuclease PARN. We show now that the miR-21-5p tailing-and-trimming pathway is controlled by miR-4728-3p-mediated downregulation of PAPD5 in *HER2*-amplified tumours. Aberrant expression of miR-21-5p has been implicated in cancer and cardiovascular disease and is associated with oncogenic processes such as epithelial-to-mesenchymal transition (EMT), cell cycle control, apoptosis, and metastasis (reviewed by Kumarswamy *et al*.^11^). The tumorigenic role of miR-21-5p is exerted through downregulation of various tumour suppressor genes; regulation of PTEN by miR-21-5p leads to induction of protein kinase B (Akt) signalling and has been associated with resistance against HER2-targeted therapies^12^. Cells resistant to trastuzumab treatment were found to overexpress miR-21-5p and ectopic expression of miR-21-5p also conferred resistance *in vitro*, an effect that was reversed by overexpression of a *PTEN* gene lacking target sites for miR-21-5p^12^.

In summary, we show that the HER2 receptor and miR-4728-3p contribute to carcinogenesis in a cooperative but independent manner; while HER2 signalling induces transcription of mir-21, miR-4728-3p contributes to the oncogenic effect by maintaining high steady-state levels of active miR-21-5p. This new oncogenic function of the *HER2* locus indicates that targeting only the HER2 receptor, the cornerstone of treatment of cancers that overexpress HER2, may not be sufficient for complete treatment of HER2-postive cancer.

## Results

### miR-4728-3p activity in HER2-positive cells regulates PAPD5

In a previous study^6^, we observed that miR-4728-3p was the main mature product of the *HER2*-encoded mir-4728 precursor. Analysis of next-generation sequencing data provided by the YM500 database version 2^13^ confirmed that expression of miR-4728-3p far exceeded -5p in cancer samples (Supplementary Fig. S1). We therefore focused on miR-4728-3p and selected two HER2-positive breast cancer cell lines, SK-BR-3 and BT-474, to block miRNA function by transfecting 2′-*O*-methyl-modified antisense oligonucleotides (ASOs). To verify the action of this treatment we cloned a 3′ untranslated region (UTR) with perfect complementarity to miR-4728-3p downstream of a firefly luciferase gene in a reporter vector. As expected, transfection of this vector showed that the presence of a miR-4728-3p target site in the 3′ UTR reduced luciferase activity through the action of endogenous miR-4728-3p and that co-transfection with miR-4728-3p ASO reverted this repression (Supplementary Fig. S2a). Global effects were then investigated by gene expression analysis 48 and 96 hours after ASO transfection. To confirm the specificity of the ASO treatment at global level we performed Gene Set Enrichment Analysis (GSEA). This analysis showed significant enrichment of TargetScan-predicted miR-4728-3p targets among upregulated genes at both time points in SK-BR-3 (FDR < 0.001 and 0.035, respectively, see Supplementary Fig. S2b), again confirming the experimental approach. The number of differentially expressed genes was considerably smaller in BT-474 compared to SK-BR-3 upon miR-4728-3p ASO treatment (125 and 412 genes at 48 h, respectively; cut-off log_2_ fold change ± 0.5 and adjusted *P* < 0.05).

**Figure 1:**
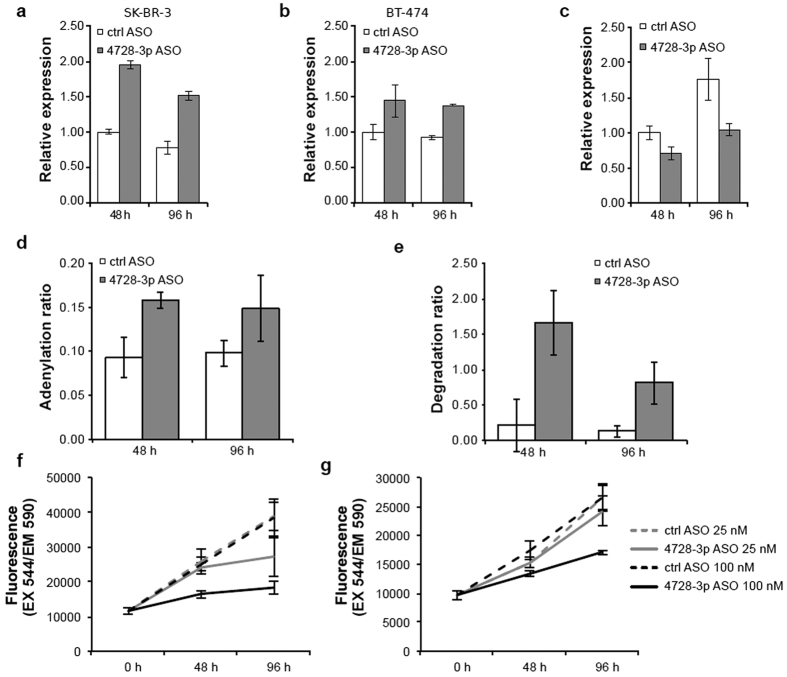
Blocking miR-4728-3p upregulates PAPD5, which mediates degradation of miR-21-5p. Expression of *PAPD5* mRNA increased at both 48 and 96 h after blocking of miR-4728-3p by transfection of a 2′-*O*-methyl-modified antisense oligonucleotide in the HER2-positive breast cancer cell lines SK-BR-3 (**a**) and BT-474 (**b**). In SK-BR-3, the expression of miR-21-5p decreased (**c**) while the adenylation (**d**) and degradation (**e**) ratios increased. Blocking miR-4728-3p specifically decreased proliferation in both SK-BR-3 (**f**) and BT-474 (**g**) cells in an alamarBlue assay. Real-time qRT-PCR results (**a–c**) are shown as mean ± s.e.m. for n = 3 replicates. Adenylation and degradation ratios from sequencing (**d,e**) are shown as mean ± s.d. for n = 3 replicates. The proliferation curves show mean background-corrected fluorescence ± s.d. for n = 4 replicates.

Among the top upregulated genes we found the non-canonical poly(A) polymerase *PAPD5* in both SK-BR-3 and BT-474 cells (log_2_ fold change 0.94, adjusted *P* = 7.80 × 10^−9^ and log_2_ fold change 0.53, adjusted *P* = 0.0018, respectively). This is interesting in light of our recent report where we show that the oncomiR miR-21-5p is subjected to 3′ adenylation by PAPD5^10^. Upregulation of *PAPD5* in HER2-positive breast cancer cell lines treated with miR-4728-3p ASO was confirmed by real-time qRT-PCR for both time points with a doubling of mRNA abundance 48 hours after ASO transfection in SK-BR-3 (Fig. 1a). Although differential expression was more modest in BT-474, we observed a clear trend of *PAPD5* upregulation upon miR-4728-3p blocking (Fig. 1b). To confirm that the observed effect on *PAPD5* was mediated by miR-4728-3p we repeated the ASO treatment in HeLa cells that do not express mir-4728 and *PAPD5* levels remained unchanged (Supplementary Fig. S3a). TargetScan lists one predicted target site for miR-4728-3p in the *PAPD5* 3′ untranslated region (UTR), suggesting a putative direct link, although repeated assays failed to show consistent functionality for this target site (data not shown).

### Blocking miR-4728-3p leads to downregulation of miR-21-5p and inhibition of cell proliferation

We then investigated whether miR-4728-3p may affect the PAPD5-mediated regulation of miR-21-5p. As previously noted, the most prominent miR-21-5p isoform produced by Dicer is a 23-nt isomiR that carries a templated cytosine at the 3′ end not present in the 22-nt miR-21-5p sequence registered in miRBase. This isomiR is called miR-21-5p + C and its 3′ end is the preferred substrate of PAPD5^10^. We first assessed miR-21-5p levels independently of isoform by real-time qRT-PCR in SK-BR-3 cells and total miR-21-5p levels were reduced when blocking miR-4728-3p (Fig. 1c). GSEA of predicted target genes for miR-21-5p from TargetScan in the microarray data also confirmed significant enrichment of targets among upregulated genes in SK-BR-3 upon blocking of miR-4728-3p at 48 and 96 h (FDR = 0.0014 and 0.0011, respectively, see Supplementary Fig. S4).

HER2 overexpression has been reported to increase the stability of the Microprocessor complex and the efficiency of Dicer cleavage of growth-promoting miRNAs^14^. Also, PARN has been shown to be involved in miRNA processing^15^. With this in mind we investigated whether changes in the miRNA processing machinery are the cause for the specific downregulation of miR-21-5p. We reasoned that if miR-4728-3p affected PAPD5/PARN tailing-and-trimming, this should only change the levels of miR-21-5p while miR-21-3p and other parts of the primary mir-21 transcript should remain unchanged. If, however, the downregulation of miR-21-5p were to be caused by aberrant processing, all parts of pri-mir-21 should change proportionally. We sequenced the small RNA fraction of miR-4728-3p ASO-treated SK-BR-3 cells in biological triplicate and evaluated the abundance of pri-mir-21 fragments in relation to miR-21-5p. As a proxy for changes in maturation we compared fragments upstream of 5p, the pre-miR loop, mature 3p and fragments downstream of 3p. We found that neither miR-21-3p nor any of the pri- or pre-miRNA fragments were affected by blocking miR-4728-3p and that the observed downregulation was specific for mature miR-21-5p (Supplementary Fig. S3b). We conclude that it is highly unlikely that differences in Drosha and Dicer processing, which would affect both mature miRNAs, could cause the observed effect on miR-21-5p.

To investigate the involvement of the PAPD5/PARN pathway, we then calculated miR-21-5p adenylation and degradation ratios in the sequencing data as described by Boele *et al*.^10^. Briefly, the adenylation ratio is calculated as the expression of adenylated miR-21-5p + C (24-mer miR-21-5p + CA) divided by miR-21-5p + C (23-mer), while the degradation ratio is calculated as miR-21-5p (22-mer) divided by miR-21-5p + C. Corresponding to the increase in PAPD5, we observed increased 3′ adenylation of miR-21-5p + C upon treatment with miR-4728-3p ASO (Fig. 1d). The degradation ratio was also elevated as expected if de-repression of tailing-and-trimming by PAPD5 and PARN is taking place (Fig. 1e). This confirms a role for miR-4728-3p in the regulation of miR-21-5p through the PAPD5/PARN pathway.

Up-regulation of miR-21-5p promotes proliferation in many different cell types^16^,^17^,^18^,^19^. To test if this was also the case upon blocking of miR-4728-3p we assayed the rate of proliferation of SK-BR-3 and BT-474 with alamarBlue after transfection with ASOs. As shown in Fig. 1f,g, blocking miR-4728-3p resulted in a significant decrease in proliferation rate in both cell types. This effect was not observed in HER2-negative MCF10A cells that do not express mir-4728 (Supplementary Fig. S5). Similar results were also obtained from HER2-negative MCF7 cells. These results indicate that this pro-proliferative process is restricted to HER2-positive cells where miR-4728-3p expression is up-regulated.

We noted that the miR-4728-3p ASO microarray data showed significant accumulation of genes involved in metabolism and oxidative phosphorylation and alamarBlue works as an indirect indicator of cell viability by measuring metabolic activity based on reduction of Resazurin to Resorufin by NAD(P)H dehydrogenase in mitochondria. We therefore wanted to exclude a metabolic change rather than a decrease in the rate of proliferation so we also counted cells under the microscope and confirmed that the observed reduction was due to a decline in cell number (Supplementary Fig. S6).

### miR-4728-3p controls miR-21-5p expression and cell proliferation independently of HER2 receptor signalling

**Figure 2:**
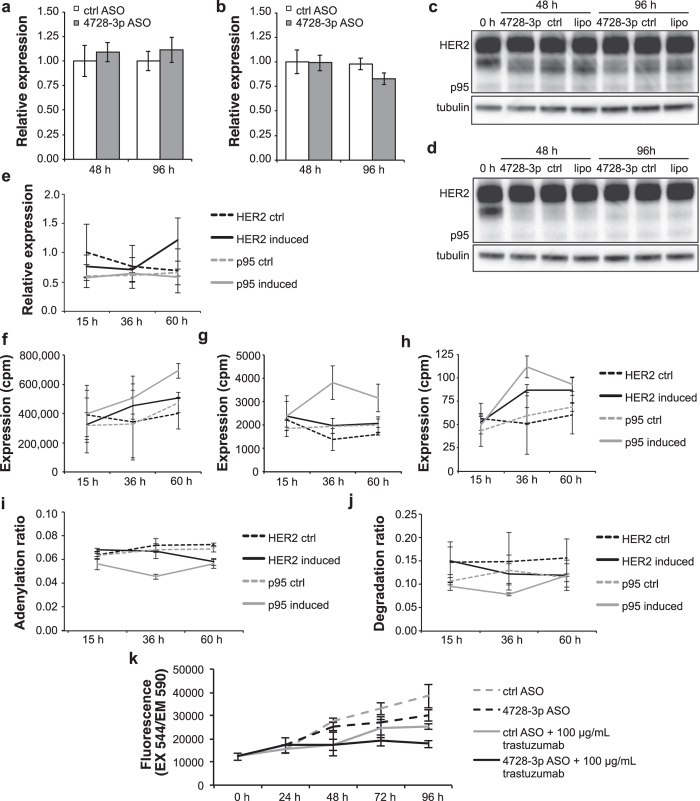
Blocking miR-4728-3p does not interfere with expression of HER2 and overexpression of full-length HER2 or a truncated, intracellular form (p95) increases transcription of mir-21. *HER2* mRNA levels remained constant upon treatment of SK-BR-3 (**a**) and BT-474 (**b**) cells with the miR-4728-3p antisense oligonucleotide. Transcript levels were quantified with real-time qRT-PCR using primers spanning the intron that encodes mir-4728. Western blotting confirmed that HER protein expression was also unchanged in SK-BR-3 (**c**) and BT-474 (**d**) cells. Contrast was increased to visualise p95-HER2. Tubulin is shown as a loading control. (**e**) As expected, expression of *PAPD5* mRNA was not affected by overexpression of cDNA clones of HER2 or p95-HER2 lacking the intronic miR-4728-3p in the HER2-negative breast cancer cell line MCF7. (**f**) Expression of mature miR-21-5p increased upon overexpression of HER2 and, more strongly, of p95-HER2. Also other parts of mir-21 were induced, including miR-21-3p (**g**) and the 5′ part of pri-mir-21 (**h**), indicating an effect at the level of transcription. Adenylation (**i**) and degradation (**j**) ratios remained largely unchanged. (**k**) Combined treatment with trastuzumab and a miR-4728-3p antisense oligonucleotide significantly decreased proliferation in SK-BR-3 cells compared to treatment with trastuzumab alone. Real-time qRT-PCR results (**a,b,e**) are shown as mean ± s.e.m. for n = 3 replicates. Sequencing results (**f–j**) are shown as mean ± s.d. for n = 2 replicates. The proliferation curves show mean background-corrected fluorescence ± s.d. for n = 4 replicates.

Since mir-4728 has been suggested to regulate factors downstream of HER2^9^, we wanted to test if HER2 signalling could control PAPD5 and thus cause the observed downregulation of miR-21-5p upon blocking of miR-4728-3p. MicroRNA-4728 is a 5′ tailed half-mirtron with its 3′ end coinciding with the 5′ splice site of the *HER2* exon 24 (NM_004448.3). We first investigated whether blocking the miRNA with ASOs could interfere with *HER2* splicing. Real-time qRT-PCR analysis showed that *HER2* mRNA levels remained unchanged upon miR-4728-3p ASO treatment in the two cell lines (Fig. 2a,b). Furthermore, a western blot for HER2 showed that also protein levels were unaffected by miR-4728-3p ASO treatment (Fig. 2c,d).

Approximately one third of HER2-positive tumours express C-terminal fragments (CTFs) of the HER2 protein generically called p95-HER2^20^,^21^. These fragments lack the extracellular domain of the HER2 receptor and are in theory resistant to trastuzumab treatment, while they respond to tyrosine kinase inhibitors such as lapatinib. In western blot analyses of ASO-treated BT-474 and SK-BR-3, C-terminal fragments of HER2 were detectable at low levels but unchanged. To further investigate any possible effect of the HER2 protein on the tailing-and-trimming of miR-21-5p we overexpressed full-length HER2 as well as p95-HER2^22^ in HER2-negative MCF7 breast cancer cells. The transfected cDNA clones produce the respective HER2 variant, but not the intronically encoded miR-4728-3p, allowing us to functionally separate the effects of miRNA and host gene. As shown in Fig. 2e, *PAPD5* levels were not influenced by overexpression of either full-length or p95-HER2. We confirmed the transcriptional induction of mir-21 by full-length HER2 and observed a similar, if not stronger, activation upon overexpression of p95-HER2. Not only miR-21-5p, but also miR-21-3p and other parts of the mir-21 precursor increased upon expression of p95 and full-length HER2 (Fig. 2f–h). The induction of both miR-21-5p and miR-21-3p was verified by real-time qRT-PCR.

To uncouple HER2-mediated transcriptional induction from PAPD5-mediated regulation of miR-21-5p we calculated the adenylation and degradation ratios for miR-21-5p after HER2 and p95-HER2 induction. Neither ratio was affected by induction of either HER2 construct, excluding the involvement of HER2 signalling in the PAPD5-mediated regulation of miR-21-5p (Fig. 2i,j). In conclusion, the *HER2* locus increases miR-21-5p levels through the cooperative action of the growth factor receptor (transcriptionally) and its encoded miRNA (stabilisation). Moreover, this shows that the PAPD5-mediated trimming pathway is independent of HER2 receptor signalling, suggesting that the pro-proliferative action of miR-4728-3p on HER2-positive cells cannot be targeted by anti-HER2 drugs. To test this idea we transfected miR-4728-3p ASOs in SK-BR-3 cells again, but this time in combination with trastuzumab. This double treatment lead to a significant reduction in proliferation compared to trastuzumab alone (Fig. 2k), indicating that current anti-HER2 therapy directed solely against the receptor may not be enough to block the complete oncogenic capacity encoded by the *HER2* locus.

### HER2-positive tumours have reduced adenylation/degradation and increased expression of miR-21-5p

**Figure 3:**
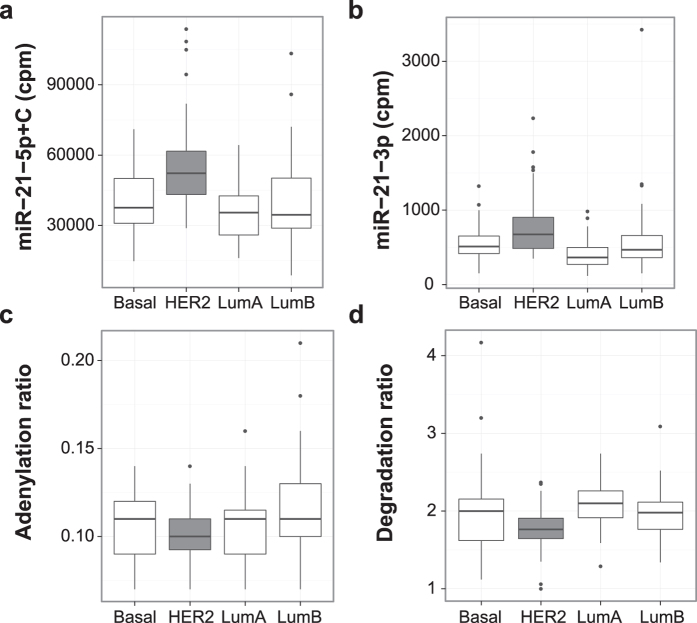
Breast tumours overexpressing HER2 have increased expression of mir-21 and decreased degradation of miR-21-5p. Breast tumours belonging to the HER2 subtype have higher expression of both miR-21-5p (**a**) and miR-21-3p (**b**) mature miRNAs compared to other molecular subtypes. Data is expressed as counts per million reads (cpm). In the SCAN-B data, the HER2 subtype also exhibited significantly decreased degradation (**d**), but not adenylation (**c**) of miR-21-5p.

Finally, to study if the mechanisms identified in these cell line experiments are also present in breast tumours we used samples included in the population-based breast cancer project SCAN-B^23^. This analysis may be complicated by the fact that miR-21-5p has a dynamic expression in different cell types within the tumour^24^ while the tumour data represents bulk tumour RNA with varying percentages of cancer cells. We selected 186 breast tumour samples, produced small RNA sequencing data from the extracted total RNA and used poly(A)-positive mRNA-sequencing data to classify samples into the intrinsic subtypes according to the expression of genes included in the PAM50 classifier. To attain more robust results we also analysed breast tumour data from The Cancer Genome Atlas (TCGA)^25^. We reasoned that if miR-4728-3p acts in concert with HER2 to increase the level of miR-21-5p, the latter should be up-regulated in HER2-like tumours compared to other breast cancer subtypes, while the adenylation and degradation ratios should decrease. Analysis of the SCAN-B data showed that expression of miR-21-5p and miR-21-3p were significantly higher in tumours belonging to the HER2-like subtype *vs* other subtypes (*P* = 9.56 × 10^−9^ and *P* = 1.40 × 10^−7^, respectively, Student’s t-test) (Fig. 3a,b) and in accordance with our results, miR-21-5p degradation ratios were also lower in the HER2-like subtype *vs* other subtypes (Fig. 3d) (P = 2.66 × 10^−5^, Student’s t-test). Furthermore, adenylation ratios were lower in the HER2-like subtype although the difference was not statistically significant (*P* = 0.18, Student’s t-test). In the TCGA data expression of miR-21-5p and miR-21-3p were also significantly higher in tumours belonging to the HER2-like subtype *vs* other subtypes (Supplementary Fig. S7a,b, *P* = 5.11 × 10^−3^ and *P* = 5.02 × 10^−4^, respectively, Student’s t-test), while the miR-21-5p adenylation ratio was significantly lower in HER2-like tumours (Supplementary Fig. S7c, *P* = 0.0013, Student’s t-test). The degradation ratio did not differ significantly in the TCGA data. Altogether the association between mir-21 expression and the HER2 subtype in these two independent tumour data sets is in agreement with our experimental results.

## Discussion

Non-templated 3′ modification of mature miRNAs by nucleotidyl transferases is a common event in animal cells^26^,^27^. Both extent and type of 3′ end additions vary widely between miRNAs and, where the functional consequences have been characterised, they do not seem to follow a general rule. For instance, 3′ terminal adenylation stabilises miR-122^28^ in what appears to be competition between the poly(A) polymerase PAPD4 (GLD2) and PARN^29^, while two of our groups recently described a tailing-and-trimming pathway for regulation of miR-21-5p abundance where the 3′ end of miR-21-5p is adenylated by PAPD5, marking the miRNA for 3′-to-5′ trimming by PARN^10^. Here we show that regulation of miR-21-5p by this pathway is controlled by the *HER2*-encoded miRNA mir-4728. We also demonstrate that inhibition of miR-4728-3p results in a significant decrease in cell proliferation, implying that this miRNA contributes to the oncogenic activity of the *HER2* locus by sustaining proliferation through inhibition of miR-21-5p degradation. Since the miRNA is normally expressed at low levels in most tissues, this regulatory mechanism cannot be assumed to be widely active in human organs, but it is induced in tumours with amplification of the *HER2* locus. This observation may have important clinical implications; since e.g. PTEN is a direct target of miR-21-5p and has been associated with trastuzumab resistance in HER2-positive breast cancer cells^12^. Although the role of miR-21-5p as sole mediator of trastuzumab resistance via PTEN regulation has been challenged^24^, it seems that at least part of the drug insensitivity can be attributed to miR-21-5p function. Regardless of whether it occurs exclusively through miR-21-5p, our work uncovered an oncogenic role for miR-4728-3p in sustaining proliferative signalling in a pathway that is independent of the HER2 receptor. This implies that although large efforts are made to improve anti-HER2 therapy, complete inhibition of the carcinogenic signals encoded by the locus cannot be achieved by only targeting the transmembrane receptor. This fact may well contribute to the fact that many patients are refractory to anti-HER2 treatment^30^. Since mir-4728 piggybacks transcription of *HER2* and, as shown above, the two genes work together in an orchestrated manner, blocking HER2 signalling exclusively could unbalance some functions of mir-4728. It is tempting to speculate that mir-4728 might be involved in some of the adverse effects of trastuzumab such as cardiotoxicity because of the well-characterised association between miR-21-5p and heart failure^31^,^32^. The effect of co-targeting mir-4728 on alleviating some of these issues as well as blocking its pro-proliferative action would be interesting to study. Since mir-4728 is lowly expressed in most tissues and amplification or overexpression of *HER2* increase the cellular concentration above a functional threshold^8^, inhibition of mir-4728 and the mir-4728/PAPD5/miR-21-5p circuit could act as an anti-HER2 cancer therapy that is selectively active in HER-positive cancer cells.

**Figure 4:**
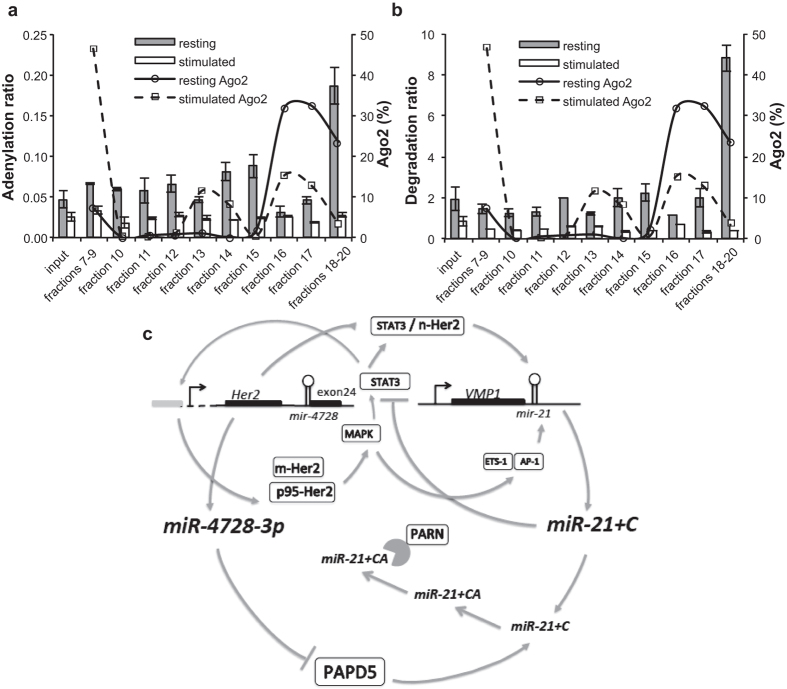
Resting mouse T cells display increased adenylation and degradation of miR-21-5p in low molecular weight protein fractions inactive in regulation of gene expression and a model summarizing the oncogenic circuit linking HER2, miR-4728-3p and mir-21. Reanalysis of small RNA sequencing data for protein fractions from murine T cells from La Rocca *et al*.^33^ revealed increased adenylation (**a**) and degradation (**b**) ratios in resting compared to stimulated T cells. Enrichment is particularly pronounced in the very low molecular weight fractions that do not contain the bulk of AGO2 protein (fractions 16 and 17). (**c**) In summary, mir-4728 is co-expressed with HER2 to produce the mature miRNA miR-4728-3p, which downregulates PAPD5. Reduced adenylation of miR-21-5p by PAPD5 prevents PARN-mediated degradation of the miRNA. Signalling by HER2 and p95-HER2 induces the MAPK1/2 pathway, which activates transcriptional regulators including STAT3, ETS-1, and AP-1, in turn increasing the transcription of mir-21. STAT3 is also regulated by miR-21-5p in a negative feedback loop. m-HER2, membrane-bound HER2; n-HER2, nuclear HER2.

The importance of keeping miR-21-5p under tight control is supported by the fact that we could not identify any other miRNA that appeared to be adenylated by PAPD5. We have also previously observed that regulation of miR-21-5p by PAPD5 and PARN is disrupted in highly proliferative tissues^10^, suggesting that degradation of miR-21-5p is restricted to cells in a state of quiescence. This idea is supported by data produced by Thompson and co-workers^33^,^34^, who showed that miRNAs in quiescent mouse cells associate with inactive, low molecular weight Argonaute complexes which lack essential components for miRNA-directed repression such as the GW182 proteins. These complexes are suggested to be reservoirs of inactive mature miRNAs that can be re-activated into high molecular weight (active) complexes upon mitogenic signalling. Reanalysing their data with focus on tailing-and-trimming of miR-21-5p we found that adenylation and degradation ratios increased in resting cells. The highest peak of adenylation/degradation was detected in the size fraction just below the ones containing the bulk of AGO2 protein (97 kDa) (Fig. 4a,b) coincident with the expected sizes of PAPD5 (63 kDa) and PARN (74 kDa). By contrast, stimulated cells showed no specific enrichment in any fraction. In concordance with our previous results, no other miRNA displayed an adenylation profile similar to miR-21-5p in the Thompson *et al*. data, confirming that this is likely a miR-21-5p-specific pathway and indicating the importance of keeping it under strict control.

In summary, the HER2 growth factor receptor induces a signalling cascade that increases transcription of mir-21, while miR-4728-3p blocks PAPD5, a negative regulator of miR-21-5p stability. Together they act to increase the levels of the oncomiR miR-21-5p and promote cell proliferation. The proposed regulatory circuit is depicted in Fig. 4c. Stimulation of the 185 kDa HER2 protein in breast cancer cells activates mir-21 transcription by induction of transcription factors including ETS-1^35^ and AP-1^36^ or through STAT3^37^. STAT3 also acts as a transcriptional activator of HER2 by binding to a response element in the *HER2* promoter and recruits nuclear HER2^38^ as a coactivator to regulate the transcription of mir-21^39^. Furthermore, miR-21-5p targets STAT3, leading to its downregulation in a negative feedback loop^40^. Interestingly, STAT3 mRNA levels increased upon blocking of miR-4728-3p in our experiments. This regulatory circuit establishes a new oncogenic role for the *HER2* locus through the intronically encoded miRNA mir-4728 that is not currently being targeted by any anti-HER2 therapy.

## Material and Methods

### Cell culture and transfections

All cell lines were purchased from ATCC and used at low passage numbers. Cells were cultured as reported previously^41^. MCF7 Tet-Off cells (BD Biosciences) expressing inducible full length or p95-HER2 were established as described^22^ and maintained in medium containing 1 μg/ml doxycycline (Sigma). Antisense oligonucleotides (IDT DNA Technologies) contained 2′-*O*-methyl modifications and are listed in Supplementary Table 1. Transient transfections were performed using Lipofectamine 2000 (Life Technologies) following the manufacturer’s instructions with 25 nM (SK-BR-3) or 100 nM (BT-474) antisense oligonucleotide, as indicated. AlamarBlue (Invitrogen) proliferation assays were performed according to the manufacturer’s instructions, using a FLUOstar Omega (BMG LABTECH) to measure fluorescence (excitation 544 nm, emission 590 nm) after 2 h incubation.

### Luciferase assay

For luciferase assays, two DNA oligonucleotides corresponding to a perfectly complementary target site for miR-4728-3p were phosphorylated, annealed and ligated between the SacI and SalI sites of the pmirGLO plasmid (Promega). Luciferase assays were performed with the Dual-Luciferase Reporter Assay System (Promega) on a FLUOstar OMEGA Microplate Reader (BMG LABTECH) at 24 h after transfection of SK-BR-3 cells with 10 ng plasmid and 25 nM ASO in 96-well plates. Luminescence readings for the firefly target site reporter gene were normalised to the signal from the Renilla reporter gene and the negative control-treated empty vector.

### Microarray expression analysis

RNA was extracted with TRIZOL (Life Technologies) according to the manufacturer’s instructions. RNA quantity and quality were assessed with NanoDrop ND 1000 spectrophotometer (NanoDrop Tech) and Bioanalyzer (Agilent) before analysis on HumanHT-12 v4.0 Expression BeadChips (Illumina). All data were imported and normalised using quantile normalization implemented on the Base server (http://base.thep.lu.se)^8^. Gene Set Enrichment Analysis (GSEA)^42^ with default settings was done for predicted miRNA targets based on TargetScan 6.2 predictions^43^ using RefSeq identifiers and gene lists were pre-ranked by log_2_ fold change between treatment and control.

### Western blot

Cells were harvested at indicated times on ice in RIPA buffer (10 mM Tris-HCl pH 7.4, 150 mM NaCl, 1 mM EDTA, 0.1% SDS, 1% Triton X-100, and 1% sodium deoxycholate) supplemented with complete protease inhibitor mixture tablets (Roche Diagnostics). Lysates were clarified by centrifugation and protein concentrations were determined by BCA Protein Assay kit (Thermo Scientific). Equal amounts of crude lysates were separated by SDS-PAGE on 4–12% bis-tris gels and proteins were transferred to a PVDF membrane (both Life Technologies). Membranes were then blocked and probed with HER2 (AMAB90627, Sigma) and tubulin (ab7291, abcam) antibodies according to the manufacturers’ instructions. HRP-conjugated secondary antibodies (abcam) were visualised with ECL (Santa Cruz) and staining intensity was determined using a Chemidoc MP (Bio-Rad).

### Real-Time quantitative RT-PCR

Reverse transcription and real-time qRT-PCR were performed as described, with poly(A) tailing and reverse transcription (RT) for miRNA qRT-PCRs and only RT for mRNAs^44^. Real-time qRT-PCR was performed with cDNA diluted 1:10 in SsoFast EvaGreen reagents (Bio-Rad) on a CFX96 instrument (Bio-Rad). Expression data were normalised to selected reference genes (PPIA for mRNA and U6, RN7SL, and let-7a for miRNA). Primer sequences can be found in Supplementary Table 1.

### Next-generation sequencing

Sequencing libraries were prepared with the NEBNext Multiplex Small RNA Library Prep Set for Illumina (New England Biolabs) according to the manufacturer’s instructions and sequenced on Illumina HiSeq sequencer in paired end mode for 2 × 101 cycles or on Illumina MiSeq in single-end mode for 51 cycles. Sequences were demultiplexed using Picard and aligned against hg19 using Novoalign with settings -a AGATCGGAAGAGCACACGTCT -l 14 -h -1 -1 -t 90 -g 50 -x 15 -o SAM -o FullNW -r All 51 -e 51. MicroRNA and isomiR expression were analysed using custom Perl scripts. Expression values for miR-21-5p in the TCGA dataset were retrieved from the TCGA website, while miR-21-5p + C and adenylated variant expression values were retrieved from the YM500 database^13^.

### Statistical analysis

For luciferase assays, the relative, normalised luminescence values were plotted as mean ± standard deviation (s.d.) and for real-time qRT-PCRs, the relative, normalised expression values were plotted as mean ± standard error of the mean (s.e.m.). Expression values from next-generation sequencing were normalised as counts per million reads (cpm), plotted as mean ± s.d. Differences among these values, and the miR-21-5p adenylation and degradation ratios were tested using Student’s t-test. Analysis of microarray data including GSEA is described in the separate section “Microarray expression analysis”.

## Additional Information

**How to cite this article**: Newie, I. *et al*. *HER2*-encoded miR-4728 forms a receptor-independent circuit with miR-21-5p through the non-canonical poly(A) polymerase PAPD5. *Sci. Rep.* **6**, 35664; doi: 10.1038/srep35664 (2016).

## Supplementary Material

Supplementary Information
