# Supplementary material for: HER2-encoded mir-4728 forms a receptor-independent circuit with miR-21-5p through the non-canonical poly(A) polymerase PAPD5
Source: Sci Rep. 2016 Oct 18;6:35664. doi: 10.1038/srep35664 (PMC5067774; doi:10.1038/srep35664)
Supplement: Supplementary Information [file srep35664-s1.pdf]

Supplementary Information

HER2-encoded mir-4728 forms a receptor-independent circuit with miR-21-5p through the non-canonical poly(A) polymerase PAPD5

Inga Newie[1,5,*], Rolf Søkilde[1,5,*], Helena Persson[1], Thiago Jacomasso[1], Andrej Gorbatenko[3], Åke Borg[1,2,5], Michiel de Hoon[4], Stine F. Pedersen[3] and Carlos Rovira[1,2,5]

[*]These authors contributed equally to this work

Author affiliation:

[1]Department of Clinical Sciences, Lund, Division of Oncology and Pathology, Lund University Cancer Center, Lund, Sweden;

[2]CREATE Health, Strategic Centre for Translational Cancer Research, Lund, Sweden;

[3]Department of Biology, University of Copenhagen, Copenhagen, Denmark;

[4]Division of Genomic Technologies, RIKEN Center for Life Science Technologies, Yokohama, Japan

[5]BioCARE, Strategic Cancer Research Program, Lund, Sweden

Supplementary Table S1. Sequences of 2'-*O*-methyl-modified antisense oligonucleotides and RT-qPCR primers.

Name	Sequence (5' – 3')
PPIA_F	GTCCGTCTTCTTCCTGCTG
PPIA_R	CATCTTCATCTCCAATTCGTAGG
ERBB2.exon23.24_F	CCCATCTGCACCATTGATGTC
ERBB2.exon23.24_R	GAGTCAATCATCCAACATTTGACC
PAPD5_F	TCATGCAATGGAAATGGTGT
PAPD5_R	TGGACTGTGTGGCAGAAGAG
let7a_F	GCAGTGAGGTAGTAGGTTGT
let7a_R	GGTCCAGTTTTTTTTTTTTTTTAACTATAC
RN7SL_F	GAATAGCCACTGCACTCCAG
RN7SL_R	CAGGTCCAGTTTTTTTTTTTTTTTAGAGACG
RNU6_F	CGCAAGGATGACACGCAAATTC
RNU6_R	CAGGTCCAGTTTTTTTTTTTTTTTAAAAATATGGAA
miR21C_F	GCAGTAGCTTATCAGACTGATGT
miR21C_R	TCCAGTTTTTTTTTTTTTTTGTCAAC
miR-4728-3p-AS	mC*mU*mG*mGmGmGmCmAmGmGmAmGmGmGmAmGmGmUmCmAmGmC*mA*mU*mG
Non-targeting-AS	mUmUmAmGmUmCmGmAmCmAmUmGmUmAmAmAmCmCmAmUmUmCmAmUmGmCmA

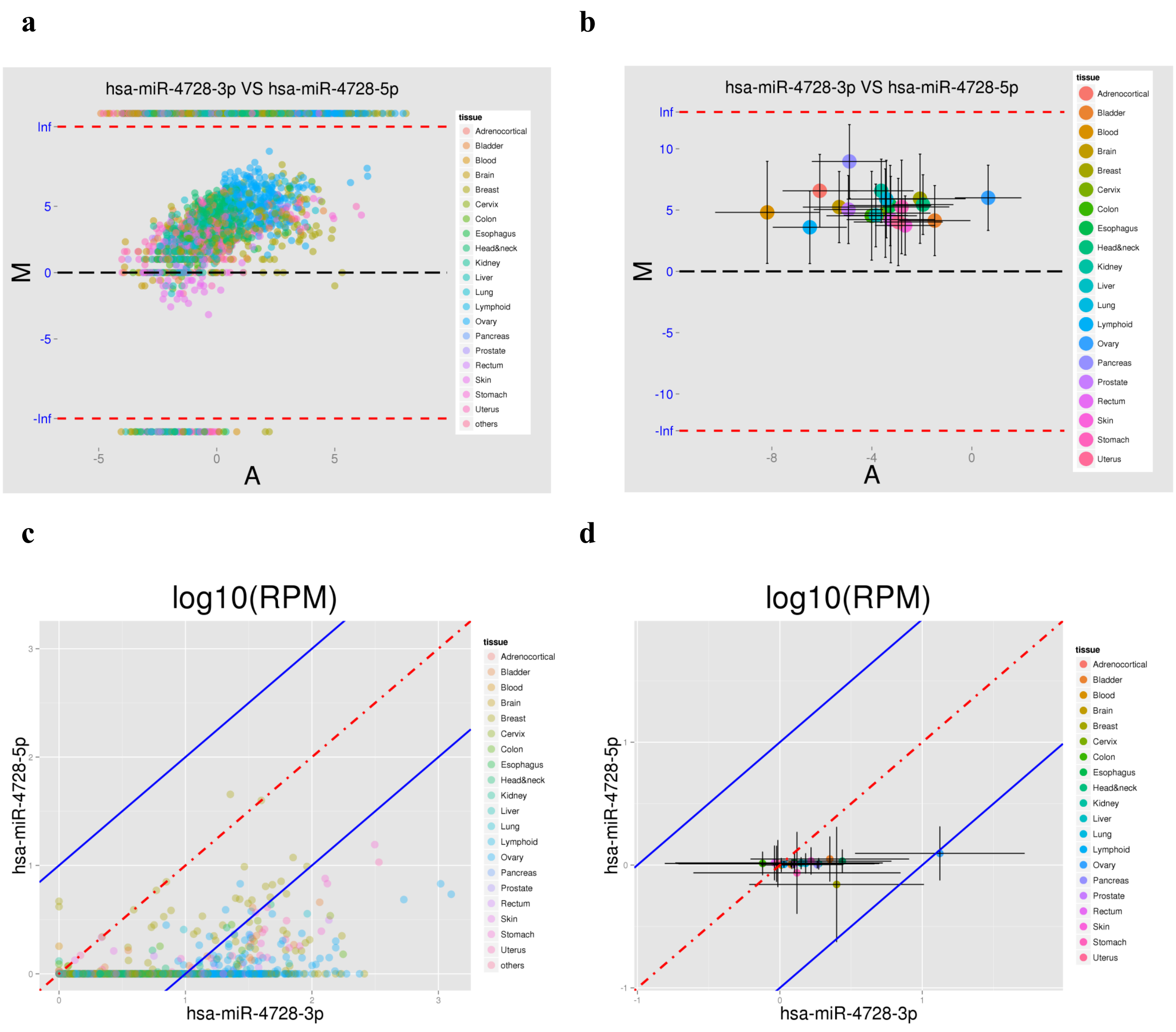

Supplementary Figure S1. The predominant mature product of mir-4728 is miR-4728-3p in most cancer types. Data are represented in MA-plots (**a-b**) and scatter-plots (**c-d**). Individual samples are plotted in (**a, c**) and (**b, d**) summarise the data in mean values with associated error bars. Most cancer samples show higher levels of miR-4728-3p compared to miR-4728-5p. Data collected from http://ngs.ym.edu.tw/ym500v2 website May 20, 2015.

a

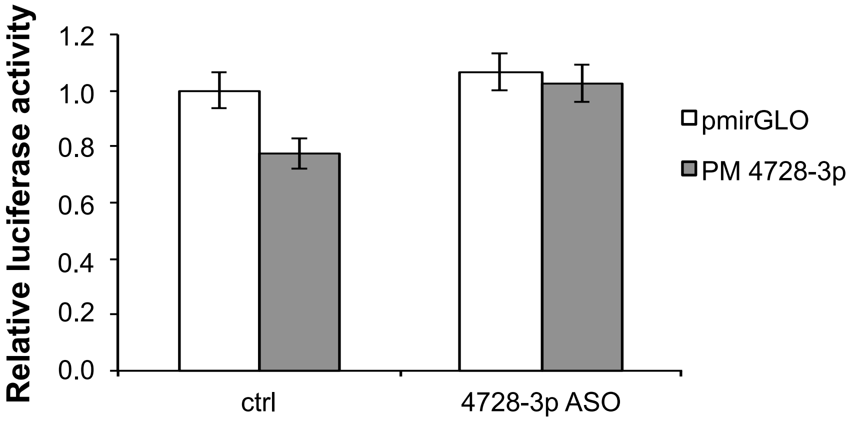

b

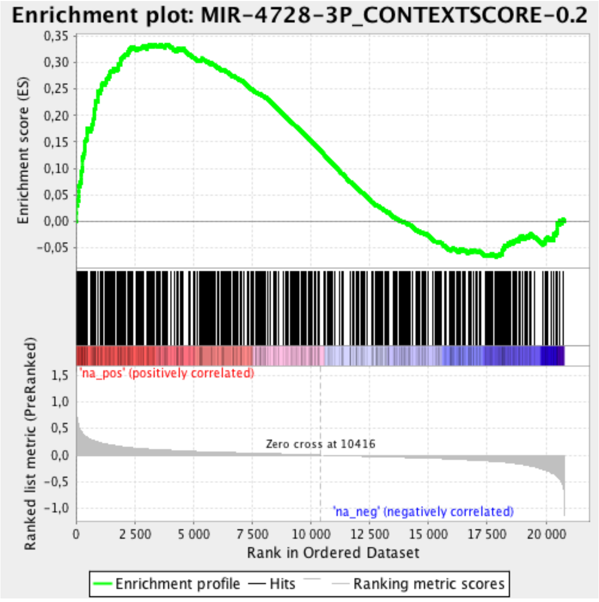

c

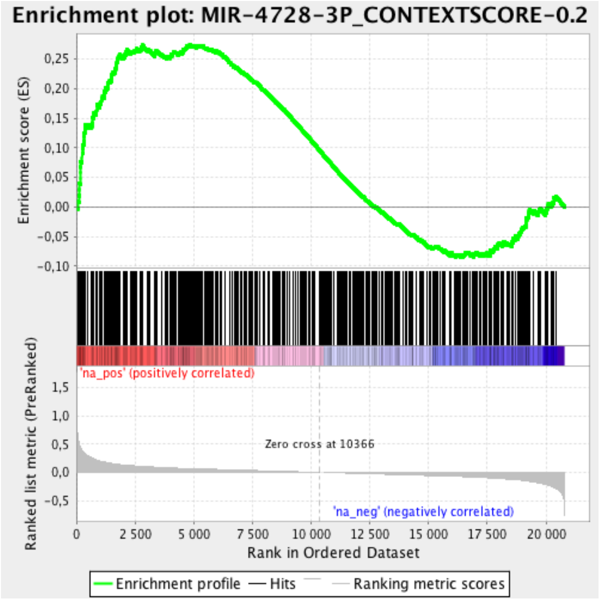

Supplementary Figure S2. Validation of the miR-4728-3p antisense oligonucleotide (ASO). a) Endogenous miR-4728-3p downregulated expression of a reporter vector carrying a perfectly complementary target site in the 3' UTR (PM 4728-3p). Repression was released upon ASO transfection. Predicted targets for miR-4728-3p were enriched among genes upregulated upon blocking of the miRNA. Gene Set Enrichment Analysis (GSEA) showed significant enrichment of predicted targets for miR-4728-3p at both (**b**) 48 h and (**c**) 96 h after transfection of a 2'-*O*-methyl-modified antisense oligonucleotide, confirming specific and efficient blocking of the miRNA.

a

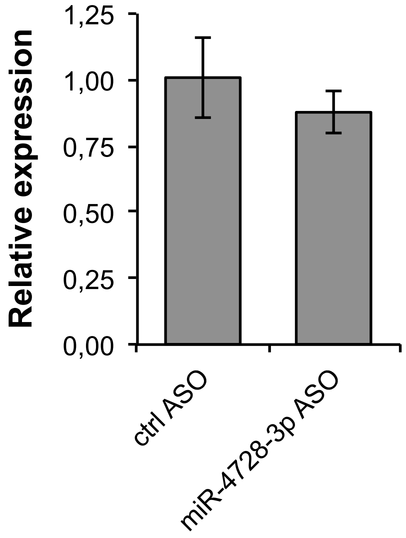

b

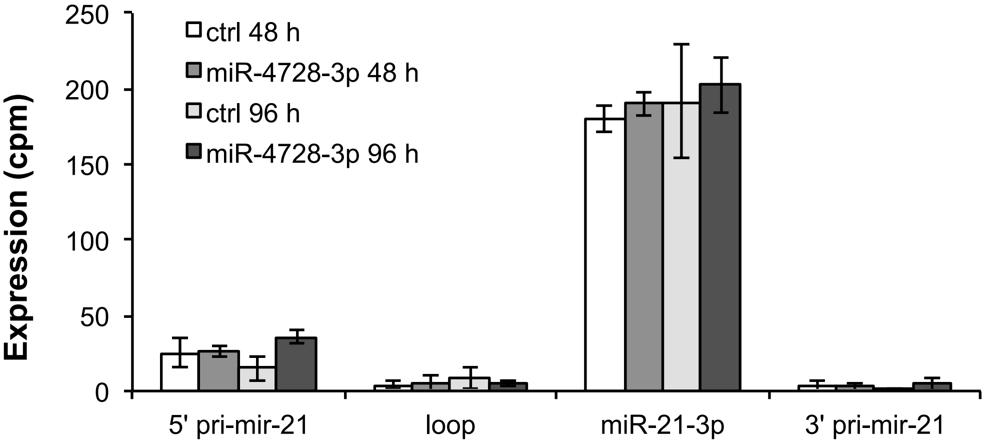

Supplementary Figure S3. Blocking miR-4728-3p does not affect PAPD5 in HER2-negative HeLa cells and the effect of PAPD5 is specific for miR-21-5p without affecting other parts of pri- and pre-mir-21. (**a**) Blocking of miR-4728-3p by transfection of a 2'-*O*-methyl-modified antisense oligonucleotide does not affect mRNA expression of *PAPD5* in HER2-negative HeLa cells which do not express miR-4728-3p. (**b**) Expression of miR-21-5p decreased specifically (**Fig. 1c**), while miR-21-3p and other parts of pri- and pre-mir-21 remained unaffected, as expected from a regulatory mechanism acting on the mature miRNA.

a

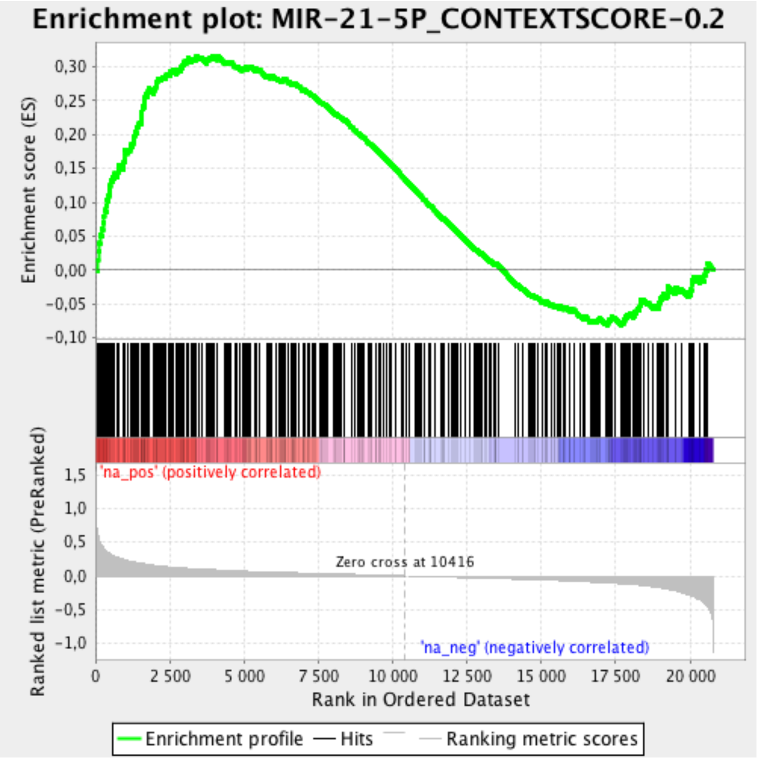

b

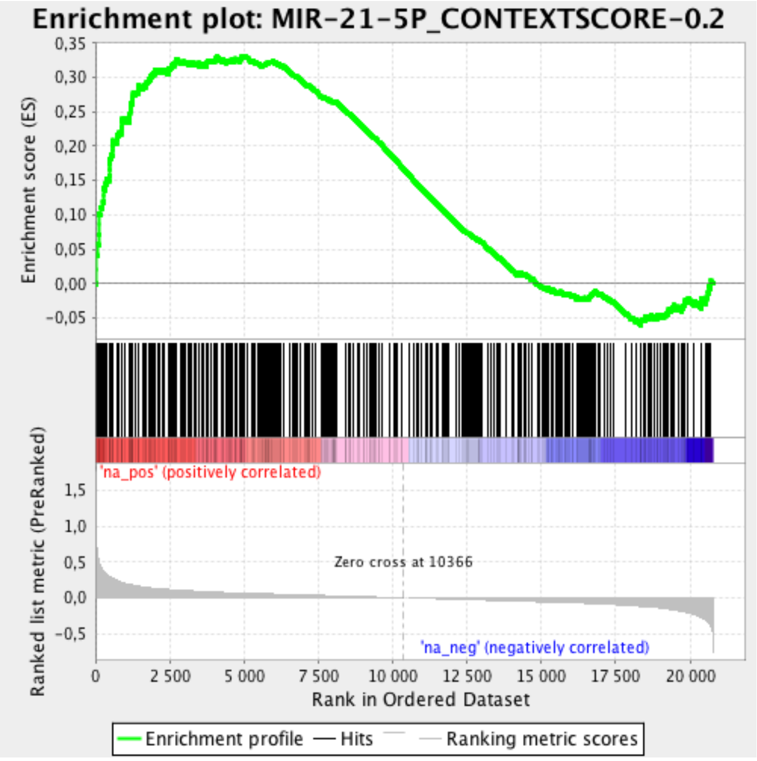

Supplementary Figure S4. Predicted targets for miR-21-5p are enriched among genes upregulated upon blocking of miR-4728-3p. Gene Set Enrichment Analysis (GSEA) showed significant enrichment of predicted targets for miR-21-5p concomitant with a decrease in total expression of the miRNA at both (**a**) 48 h and (**b**) 96 h after transfection of a 2'-*O*-methyl-modified antisense oligonucleotide against miR-4728-3p.

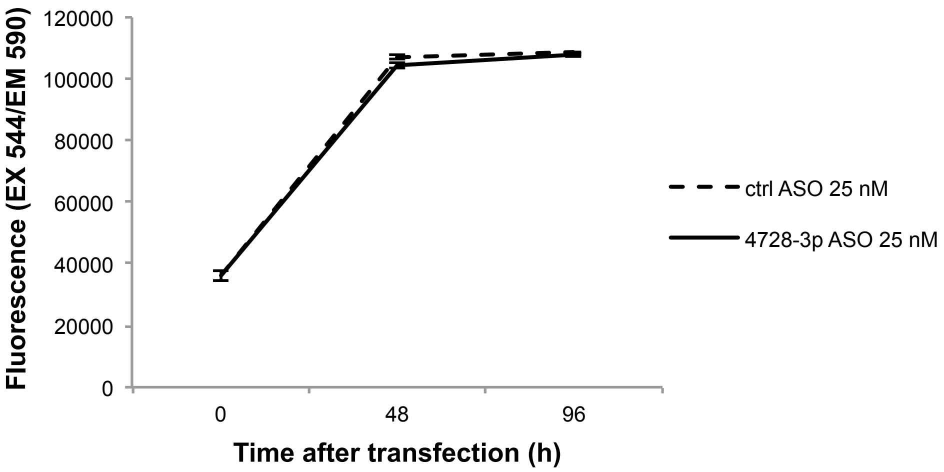

Supplementary Figure S5. Blocking miR-4728-3p does not affect proliferation in the HER2-negative breast cancer cell line MCF10A. Blocking of miR-4728-3p by transfection of 25 nM 2'-*O*-methyl-modified antisense oligonucleotide did not decrease proliferation compared to a non-targeting control oligonucleotide.

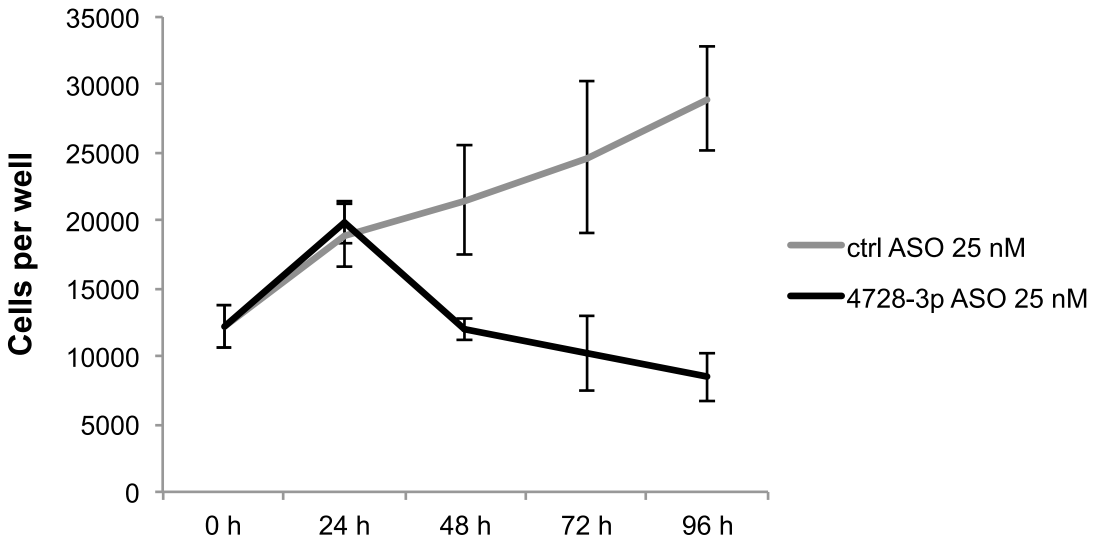

Supplementary Figure S6. Blocking miR-4728-3p decreases proliferation in the HER2-positive breast cancer cell line SK-BR-3. Blocking of miR-4728-3p by transfection of 25 nM 2'-*O*-methyl-modified antisense oligonucleotide decreased proliferation compared to a non-targeting control oligonucleotide. Cell counts obtained from manual counting are shown as mean ± s.d. for n = 4 replicates.

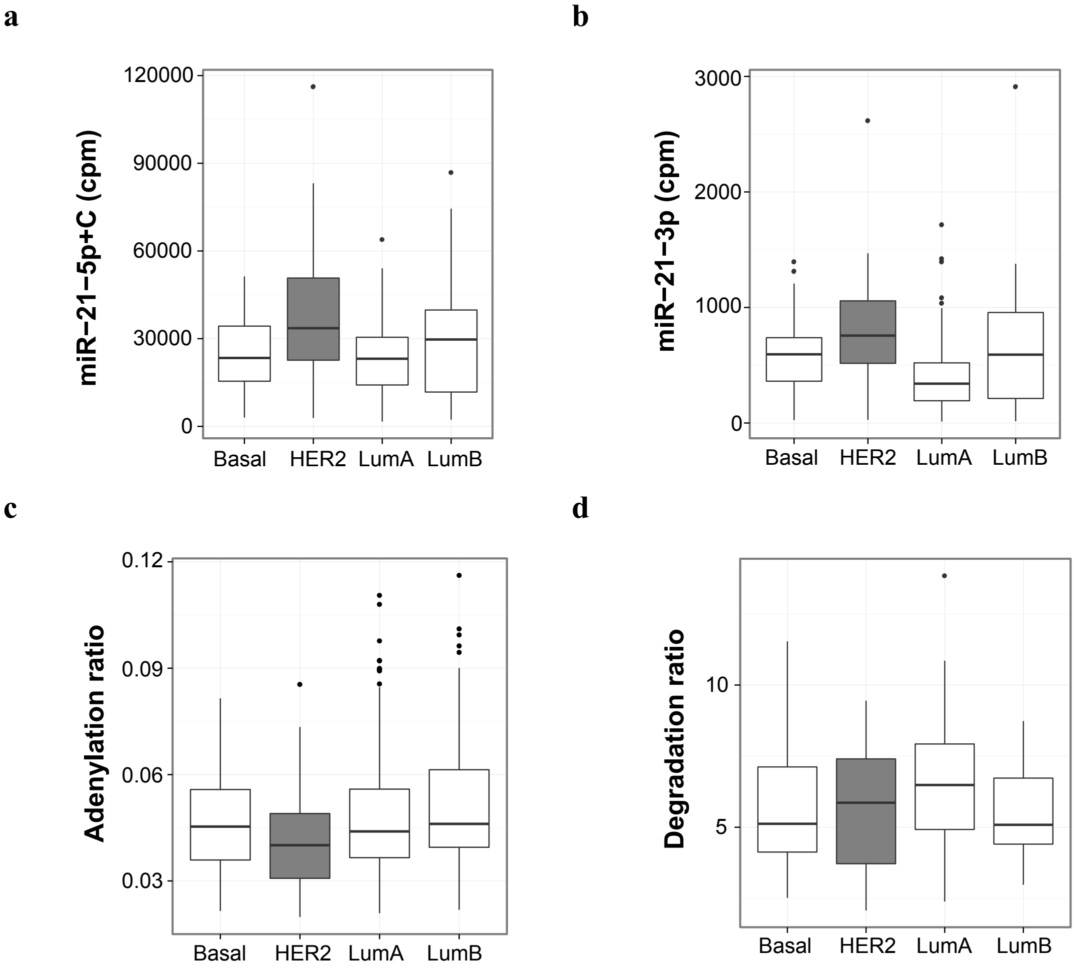

Supplementary Figure S7. Breast tumours of the HER2-like subtype have increased expression of mir-21 and decreased adenylation of miR-21-5p. Breast tumours belonging to the HER2 subtype have higher expression of both miR-21-5p (**a**) and miR-21-3p (**b**) mature miRNAs compared to other molecular subtypes. Data is expressed as counts per million reads (cpm). In the TCGA data, the HER2 subtype also exhibited significantly decreased adenylation (**c**), but not degradation (**d**) of miR-21-5p.
